# Phylogenetic typing and detection of extended-spectrum β-lactamases in Escherichia coli isolates from broiler chickens in Ahvaz, Iran

**Published:** 2016-09-15

**Authors:** Ramezan Ali Jafari, Hossein Motamedi, Elham Maleki, Reza Ghanbarpour, Mansoor Mayahi

**Affiliations:** 1 *Department of Clinical Sciences, Faculty of Veterinary Medicine, Shahid Chamran University of Ahvaz, Ahvaz, Iran; *; 2 *Department of Biology, Faculty of Science, Shahid Chamran University of Ahvaz, Ahvaz, Iran; *; 3 *Post Graduate Student, Faculty of Veterinary Medicine, Shahid Chamran University of Ahvaz, Ahvaz, Iran; *; 4 *Department of Microbiology, Faculty of Veterinary Medicine, Shahid Bahonar University of Kerman, Kerman, Iran.*

**Keywords:** Beta-lactamase, Chicken, *Escherichia coli*, Phylogeny

## Abstract

This study was conducted to reveal the phylogenetic background, to detect the genes encoding TEM, SHV and CTX-M-15 extended-spectrum β-lactamases (ESBL), and to analyze their distribution in phylo-groups of 150 *Escherichia coli* isolates from broiler chickens in Ahvaz (Southwest of Iran). Seventy- five cloacal swabs from healthy birds (fecal isolates), and 75 heart blood samples from birds with *colibacillosis* (septicemic isolates) were obtained. All isolates were phylotyped and screened for ESBL genes by polymerase chain reaction (PCR). The fecal isolates belonged to four main phylo-groups, including 41 isolates (54.67%) to A, 9 (12.00%) to B1, 5 (6.67%) to B2, and 20 (26.67%) to D. Of septicemic isolates, 37 isolates (49.33%) were classified as phylotype A, 5 (6.67%) as B1, 10 (13.33%) as B2, and 23 (30.67%) as D. In molecular analysis, a total of 72 isolates (35 fecal and 37 septicemic) were identified to harbor ESBL genes, which were distributed in phylo-groups A, B1, B2, and D. Regardless of the type of isolate, *bla*_CTX-M-15_ gene was the most common genotype, followed by *bla*_TEM_ and *bla*_SHV_ genes. This study suggests that broiler chickens in Iran are infected to ESBL genes- harboring *Escherichia coli* strains which may be spread to the food chain through fecal contamination of carcasses during slaughtering.

## Introduction

Escherichia coli is a normal inhabitant of gastro-intestinal tract of humans and animals.^1^ Phylogenetic studies have indicated that the great majority of E. coli strains can be classified into one of the four phylo-groups (A, B1, B2, and D) according to the combination of the three genetic markers: chuA, yjaA, and DNA fragment TspE_4_.C_2_ as previously described by Clermont et al.^2^ The diversity of genetic background among the phylo-groups apparently influences their antibiotic-resistance profile and growth rate,^3^ prevalence of virulence factors,^4^ and their ability to establish a population.^5^ Johnson et al. found that the strains in phylo-groups B2 and D carried more virulence factors than those in groups A and B1.^4^ Gordon and Cowling observed that the relative prevalence of phylogenetic groups among host individuals depended on the host habitat, host diet, typical body temperature, gut morphology and geographical factor. They also suggested that the competitive interactions among strains within a host may play a role as well. ^5^

In poultry, some intestinal strains referred to as avian pathogenic E. coli (APEC) may secondarily cause a local or systemic extra-intestinal infection which is responsible for significant losses and antimicrobial treatment costs.^6^^,^^7^ In the past few years, both the incidence and severity of infection with APEC isolates have increased rapidly, and it is likely to become an even greater problem in poultry industry.^8^ Antimicrobial therapy is an important tool in reducing both the incidence and mortality associated with avian colibacillosis.^9^ β-lactam antibiotics are one, if not the most important, group of antimicrobial agents used in medicine and veterinary medicine. Resistance to β-lactam antibiotics in bacterial agents is mostly mediated by β-lactamases which hydrolyze the β-lactam ring and thus inactivate the antibiotic.^1^ Up to now, more than 400 β-lactamases have been reported, and new β-lactamases continue to emerge worldwide.^10^ β-lactamases are classified on the basis of their primary structures into four molecular classes (A through D).^11^ Class B and C β-lactamases have a broader spectrum of activity, but are nearly always encoded by chromosomal genes, and hence are confined to particular bacterial species.^12^ Enzymes TEM, SHV and CTX-M belong to class A, being the predominant plasmid-mediated extended-spectrum β-lactamases (ESBLs) in Gram-negative bacteria, and can be easily transferred between and within bacterial species.^12^^,^^13^ In some cases, the presence of ESBLs in Enterobacteriaceae bacteria has been accompanied with resistance to non-β-lactam antibiotics such as fluro-quinolones and aminoglycosides.^14^ At slaughter-houses, fecal contamination of poultry carcasses could spread the resistant bacteria to the food chain, and thus transfer resistance genes to human pathogenic bacteria- which are serious menaces to public health.^15^ To our knowledge, data correlating β-lactamases in commensal and pathogenic E. coli strains from animal origin in Iran are limited. Therefore, this study was carried out to determine the phylogenetic background, to establish the prevalence of ESBL genes encoding TEM, SHV and CTX-M-15, and to analyze the phylogenetic group distribution of the resistance genes in E. coli isolates recovered from broiler chickens in Ahvaz (Southwest of Iran).

## Materials and Methods

Sample collection. Between April 2013 and March 2014, a total of 15 broiler chicken farms located around Ahvaz city (Southwest of Iran) were sampled. The birds were aged between 21 and 42 days, and had not received any antibiotic medication at least one week before sampling. From each farm, five cloacal swabs from healthy birds (totally 75 fecal isolates) and five heart blood samples from birds with overt lesions of colibacillosis (totally 75 septicemic isolates) were obtained.

Bacterial examination. Samples were plated onto MacConkey agar (Merck, Darmstadt, Germany), and incubated overnight at 37 ^°^C. One of the pink colonies from each plate was picked, streaked onto eosin methylene blue agar (Merck) plate, and incubated overnight at 37 ^°^C. Then, one colony with dark-blue center and green metallic sheen was selected from the solid medium, and identified as E. coli by using a panel of biochemical tests that included gas production and sugar fermentation reaction on triple sugar iron agar, indole production, motility, citrate fermentation, urease production, lysine decarboxylation, and methyl red Voges-Proskauer.^16^ Finally, all isolates were stored in tryptic soy broth (Merck) with 30.00% sterile glycerol (Merck) at – 70 ^°^C.

Antimicrobial susceptibility testing. The anti-microbial susceptibility of all Escherichia coli isolates was performed according to Kirby-Bauer disc diffusion method. Clinical and Laboratory Standards Institute (CLSI) guidelines were followed for inoculum standardization, medium, incubation condition, and internal quality control organism (E. coli ATCC 25922). Briefly, after adjusted in phosphate-buffered saline to 0.5 McFarland standard, the inoculum was triple streaked (a 60^°^ rotation of the round Petridish within streaks) on Mueller-Hinton agar (Oxoid, Basingstoke, UK) plate. Then, antimicrobial discs (Padtan-Teb Co., Tehran, Iran) were placed manually onto the medium by means of a sterile forceps. A combination of cefotaxime (30 µg) and ceftazidime (30 µg) with and without clavulanic acid (10 µg) was used. If the diameter of the inhibition zone in the double disc was at least five millimeters more than that in a single disc, the isolate was considered to be an ESBL producer.^17^

DNA extraction. All E. coli isolates were cultured onto nutrient agar (Merck) plates for 20 hr at 37 ^°^C. Then, five colonies were transferred to sterile distilled water in a sterile Eppendorf tube (Greiner, Austria), and boiled to prepare template DNA for PCR. The templates were stored at – 20 ^°^C.^18^

Phylogenetic group determination. According to Clermont et al., the phylogenetic group of each strain was determined by multiplex PCR of the genes chuA and yjaA and the DNA fragmentTspE_4_.C_2_.^2^ The amplification products were separated in 1% agarose gel (Cinagen, Tehran, Iran). After electrophoresis, the gel was photographed under ultraviolet light, and the strains were allocated to phylo-genetic groups A (chuA-, TspE_4_.C_2_-), B1 (chuA-, TspE_4_.C_2_+), B2 (chuA+, yjaA+) or D (chuA+, yjaA-).^2^ The primer sequences (Bioneer, Daejeon, South Korea) used in PCR, and expected size of products are presented in Table 1. Escherichia coli strain ECOR 62 was employed as positive control, and sterile distilled water as negative control.

**Table 1:** Primer sequences used to amplify phylogenetic groups and β–lactamase genes by the PCR technique

**Gene**	**Target**	**Primer**	**Product size (bp)**	**Reference**
***chuA***	__	F: GACGAACCAACGGTCAGGAT R: TGCCGCCAGTACCAAAGACA	279	Clermont *et al.*^2^
***yjaA***	__	F: TGAAGTGTCAGGAGACGCTG R: ATGGAGAATGCGTTCCTCAAC	211	Clermont *et al.*^2^
***TspE4.C2***	__	F: GAGTAATGTCGGGGCATTCA R: CGCGCCAACAAAGTATTACG	152	Clermont *et al.*^2^
***bla***_TEM_	β-lactam	F: ATAAAATTCTTGAAGACGAAA R: GACAGTTACCAATGCTTAATC	1080	Sharma *et al.*^18^
***bla***_SHV_	β-lactam	F: CACTCAAGGATGTATTGTG R: TTAGCGTTGCCAGTGCTCG	928	Sharma *et al.*^18^
***bla***_CTX-M-15_	β-lactam	F: CGCTTTGCGATGTGCAG R: ACCGCGATATCGTTGGT	550	Pitout *et al.*^19^

Detection of β-lactamase genes. Genes encoding TEM, SHV and CTX-M-15 enzymes were detected by uniplex PCR assay on genomic DNA extracted as described above. The primer sequences used for amplification of the genes are shown in Table 1.^18^^,^^19^ In PCR assay; the final 25 µL reaction mixture contained 2 mM of MgCl_2_, 1X PCR buffer, 1 U of Taq DNA polymerase (Fermentas, Waltham, USA), 50 pmol of each primer, 200 mM of each dNTPs (Fermentas) and 2 µL of template DNA. Amplification program for bla_TEM_ and bla_SHV_ was performed as initial denaturation (94 ^°^C, 3 min), 35 cycles each consisted of denaturation (94 ^°^C, 30 sec), annealing (50 ^°^C, 30 sec), extension (72 ^°^C, 2 min) and final extension (72 ^°^C, 10 min); but the program for bla_CTX-M_ and bla_CTX-M-15_ genes was initial denaturation (94 ^°^C, 5 min), 30 cycles each was comprised of denaturation (94 ^°^C, 30 sec), annealing (60 ^°^C, 30 sec), extension (72 ^°^C, 30 sec) and final extension (72 ^°^C, 10 min). The amplification products were analyzed by electrophoresis in 1% agarose gel. Two standard strains were used as positive controls: E. coli ATCC 35218 for bla_TEM_, and Klebsiella pneumonia ATCC 700603 for bla_SHV_ and bla_CTX-M-15_. Strain of E. coli ATCC 25922 was employed as negative control. Also, a 100 bp DNA marker was used in all electrophoresis for determining the PCR product size.

Statistical analysis. Statistical analyses were performed using SPSS software (version 20.0; IBM, Armonk, USA). The Chi-square and Fisher's exact tests were used to evaluate the differences between fecal and colisepticemic isolates, and the significance of differences was set at p < 0.05.

## Results

In this study, the 150 E. coli isolates belonged to four phylo-groups, including 78 isolates (52.00%) to A, 14 isolates (9.33%) to B1, 15 isolates (10.00%) to B2, and 43 isolates (28.67%) to phylo-group D (Table 2). The phylo-genetic typing of 75 fecal isolates showed that they fell into four phylo-groups: 41 isolates (54.67%) into A, 9 isolates (12.00%) into B1, 5 isolates (6.67%) into B2, and 20 isolates (26.67%) into phylo-group D. Out of 75 septicemic isolates, 37 isolates (49.33%) were classified as phylotype A, 5 (6.67%) as B1, 10 (13.33%) as B2, and 23 (30.67%) as D. The statistical analysis showed that there was no significant difference in the prevalence of phylo-groups between fecal and septicemic isolates (p > 0.05).

**Table 2:** Distribution of *Escherichia coli* isolates from healthy (n = 75) and septicemic (n = 75) broiler chickens in phylo-genetic groups.

**Groups**	**Healthy isolates (%)**	**Septicemic isolates (%)**
***chuA*** **-, ** ***TspE***_4_ ***.C***_2_ **-**	41 (54.67)	37 (49.33)
***chuA*** **-, ** ***TspE***_4_ ***.C***_2_ **+**	9 (12.00)	5 (6.67)
***chuA*** **+,** *** yjaA*** **+**	5 (6.67)	10 (13.33)
***chuA*** **+,** *** yjaA*** **-**	20 (26.67)	23 (30.67)

No significant differences were detected among the groups (*p *> 0.05).

The phenotypic screening for ESBL production detected 24 (16.00%) isolates from a total of 150 isolates as ESBL-producer; but in PCR testing, 72 (48.00%) isolates (35 fecal and 37 septicemic) were identified to carry ESBL genes. The bla_TEM_, bla_SHV_, and bla_CTX-M-15_ genes were identified in isolates recovered from both fecal and septicemic samples, but their prevalence was not influenced by the type of the sample (p > 0.05). A single resistance gene was detected in 57 (38.00%) isolates, but 15 (10.00%) isolates showed two or three resistance genes. Out of 150 tested isolates, 28 (18.67%) isolates were positive for bla_TEM_ gene, 8 (5.33%) isolates for bla_SHV_, and 52 (34.67%) isolates for bla_CTX-M-15_ (Table 3).

**Table 3:** Detailed prevalence of extended-spectrum β-lactamase genes in *Escherichia coli* isolates from healthy (n = 75) and septicemic (n = 75) broiler chickens

**Gene**	**Healthy isolates (%)**	**Septicemic isolates (%)**
***bla***_TEM_	11 (14.67)	6 (8.00)
***bla***_SHV_	0 (0.00)	1 (1.33)
***bla***_CTX-M-15_	17 (22.67)	22 (29.33)
***bla***_TEM__+_*** bla***_SHV_	1 (1.33)	1 (1.33)
***bla***_TEM__+_*** bla***_CTX-M-15_	3 (4.00)	5 (6.67)
***bla***_SHV__+_*** bla***_CTX-M-15_	2 (2.67)	2 (2.67)
***bla***_TEM__+_*** bla***_SHV__+_*** bla***_CTX-M-15_	1 (1.33)	0 (0.00)
**Total**	35 (46.67)	37 (49.33)

No significant differences were detected among the groups (*p *> 0.05).

The ESBL-positive isolates belonged to four phylo-groups A, B1, B2, and D. Out of 72 positive isolates, 32 (44.44%) were allocated to phylo-group A, 10 (13.88%) to B1, 10 (13.88%) to B2, and 20 (27.78%) to D; but in detailed explanation, the bla_TEM_ gene was not identified in phylo-group B2 of fecal isolates, and the bla_SHV_ gene was not detected in phylo-groups B1 and B2 of septicemic isolates (Table 4).

**Table 4:** Distribution of extended-spectrum β–lactamase genes in different phylo-groups of *Escherichia coli* isolates from broiler chickens.

**Type of isolates**	**Gene**	**Number of positive isolates/total isolates in each phylo-group (%)**			
		***chuA*** **-, ** ***TspE***_4_ ***.C***_2_ **-**	***chuA*** **-, ** ***TspE***_4_ ***.C***_2_ **+**	***chuA*** **+,** *** yjaA*** **+**	***chuA*** **+,** *** yjaA*** **-**
**Fecal (n = 75)**	*bla*_TEM_	7/41 (17.07)	2/9 (22.22)	0/5 (0.00)	7/20 (35.00)
	*bla*_SHV_	1/41 (2.44)	1/9 (11.11)	1/5 (20.00)	1/20 (5.00)
	*bla*_CTX-M-15_	10/41 (24.40)	5/9 (55.56)	2/5 (40.00)	6/20 (30.00)
**Total**		14/41 (34.15)	7/9 (77.78)	2/5 (40.00)	12/20 (60.00)
**Septicemic (n = 75)**	*bla*_TEM_	2/37 (5.41)	2/5 (40.00)	3/10(30.00)	5/23 (21.74)
	*bla*_SHV_	2/37 (5.41)	0/5 (0.00)	0/10 (0.00)	2/23 (8.70)
	*bla*_CTX-M-15_	16/37 (43.24)	3/5 (60.00)	6/10 (60.00)	4/23 (17.39)
**Total**		18/37 (48.65)	3/5 (60.00)	8/10 (80.00)	8/23 (34.78)

## Discussion

Escherichia coli strains are mainly assigned to four phylo-groups A, B1, B2 and D.^2^ Phylotyping analyses have shown that commensal strains usually belong to groups A and B1, whereas the extraintestinal pathogenic strains belong mainly to groups B2 and D.^20^^,^^21^ In the current study, we found the prevalence of groups A and D both in fecal and septicemic isolates, but their prevalence was not affected by the type of isolate (p > 0.05). The predominance of phylo-group A in fecal samples has been reported in previous works conducted in Iran and other countries as well.^22^^-^^25^

In contrast, Escobar‐Páramo et al. analyzing fecal strains isolated from birds, non-human mammals and humans observed the prevalence of groups D and B1 in birds, A and B1 in non-human mammals, and A and B2 in humans.^26^ The higher prevalence of phylo-groups A and D among septicemic isolates is in agreement with what have been reported previously; Rodriguez-Siek et al. divided APEC isolates from chickens into phylo-groups A (38.00%), B1 (15.00%), B2 (18.50%), and D (28.00%).^27^ Dissanayake et al. indicated that APEC strains isolated from septicemic broilers and layers were mostly distributed in phylo-groups A (71.00%), followed by D (18.70%), B2 (7.90%), and B1 (4.10%).^25^ Ghanbarpour et al. found that E. coli isolates recovered from colisepticemic broilers belonged to phylo-groups A (44.70%), B1 (21.30%), B2 (8.50%), and D (25.50%).^22^ A study on distribution of APEC strains from Japanese quails in Southeast of Iran, Salehi and Ghanbarpour identified 55.00% of the isolates as A, 18.30% as B1, 17.40% as B2 and 9.20% as phylo-group D.^28^ Hassani et al. analyzing the genomic background of E. coli recovered from broilers with colibacilosis in Northwest of Iran segregated the isolates in four phylo-groups: A (50.00%), B1 (2.80%), B2 (1.30%), and D (46.00%).^29^ Therefore, the results obtained from this study and previous works imply that E. coli strains isolated from septicemic cases are likely the typical commensals which have been become virulent by acquisition of virulence genes from pathogenic types or through random functional point mutations.^30^^,^^31^

Recent studies have demonstrated a drastic increase in the prevalence of ESBLs among bacteria of the family Enterobacteriaceae. In the current study, 48.00% of the tested isolates contained one or more ESBL genes. This result is similar to what have been reported by others though there are some contrasts which could be due to differences in type of sample, isolation and testing methods, and geographical area. In Belgium, Smet et al. identified ESBL-producing E. coli isolates in 45.00% of cloacal samples from five broiler farms.^32^ Out of 26 food samples of chicken or turkey origin analyzed in Tunisia, 7 (26.92%) samples carried E. coli with phenotype ESBL.^15^ In comparison with our results, a higher prevalence of ESBL-producing E. coli isolates (22 out of 26 farms) was observed in cloacal swabs collected from Dutch broilers. In the same study, six of 18 broiler farmers carried isolates containing ESBL genes.^33^ Also, in a study on samples recovered from healthy broiler chicken in Germany, 88.60% of carcasses and 72.50% of ceca were positive for ESBL producers, which most of them were identified as E. coli.^34^ Overdevest et al. found that 76.80% of chicken meat samples collected in the Netherlands contained ESBL-producing E. coli isolates. They also reported that 39 (69.60%) E. coli isolates recovered from rectal swabs of hospitalized patients were positive for ESBL.^35^ Extended-spectrum β-lactamase-producing Enterobacteriaceae, in particular E. coli, are isolated with increasing frequency from human and other animal samples as well. In a study performed on isolates originated from humans (n = 183), dogs (n = 77), cats (n = 11) and horses (n = 100), Schmiedel et al. found that 83.60% of the human isolates and 91.60% of the animal isolates were ESBL-producers, and concluded that multi-resistant Enterobacteriaceae could be disseminated among human and animal populations.^36^

The epidemiology of ESBL genes is changing rapidly.^13^ During the 1990s, most reports on ESBLs involved TEM/ SHVtype enzymes, but the enzymes of CTX-M type have become the most prevalent family of ESBLs among Enterobacteriaceae since their first report in 1986.^37^ In our study, bla_CTX-M-15_ was the most common genotype (34.67%), followed by bla_TEM_ (18.67%) and bla_SHV_ (5.33%), which are in accordance with previous studies. Bagheri et al. isolated 204 E. coli strains from the external and internal cavity surfaces of broiler chicken carcasses in a slaughterhouse in Kerman province (Southeast Iran), and found that 27 (13.24%) and 4 (1.96%) isolates harbored the bla_TEM_ and bla_SHV_ genes, respectively.^38^ Momtaz et al. reported a relatively high prevalence of non-β-lactam antibiotic resistance genes in 57 E. coli isolates recovered from chicken meat samples in an abattoir in Shahrekord in Iran, but could not find any positive isolates for bla_SHV_ gene.^39^ A study on the presence of ESBL genes in 22 ampicilin-resistant E. coli isolates from feces of healthy broilers revealed that 17 (77.27%) isolates were positive for bla_TEM_ gene, but none of them showed a positive reaction for bla_SHV_ gene.^40^ Out of 51 ESBL-producing E. coli isolates recovered from Belgian broilers and characterized by PCR, Smet et al. detected bla_TEM_ and bla_CTX-M_ type genes in 26 (50.98%) and 22 (43.14%) isolates, respectively, but none of the isolates carried bla_SHV_ gene.^33^ In Portugal, 38.2% of fecal samples obtained from broiler chickens in a slaughter-house were positive for ESBLs of the TEM and CTX-M groups.^41^ In the United States and Germany, the predominant ESBL subtype in human isolates was reported to be CTX-M-15.^36^^,^^42^

Regarding to the distribution of ESBL genes in different phylo-groups, the resistance genes were segregated in phylo-groups A (32 isolates), B1 (10 isolates), B2 (10 isolates) and D (20 isolates), which is in accord with other reports. For example, Bagheri et al. reported that the 27 bla_TEM_ positive E. coli isolates recovered from chicken carcasses belonged to phylo-groups: A (14 isolates), B1 (six isolates), B2 (two isolates) and D (five isolates), but all of the four bla_SHV_ positive isolates belonged to phylo-group A.^38^ In Portugal, a study on E. coli isolates recovered from raw chicken carcasses and feces of healthy chickens and swine indicated that the ESBL-positive isolates were distributed in a descending order in phylo-groups A, B1, and D.^43^ In the study performed by Slama et al. in Tunisia, the 13 ESBL-positive E. coli isolates recovered from food samples belonged to phylo-groups A (nine isolates) or D (four isolates).^15^

In conclusion, our study showed a relatively high diversity of ESBL genes among commensal and pathogenic E. coli isolates from broiler chickens in Southwest of Iran, which could be of a great concern to animal and public health. Although the use of β-lactam antibiotics in chickens is unusual, the possibility of cross-selection with other antimicrobials used in poultry (such as sulfonamides and tetracyclines) may explain this result and should be further analyzed in future.
